# Application of a Temporal Fusion Transformer and Long-Term Climate and Disease Data to Assess the Predictive Power and Understand the Drivers for Malaria and Dengue

**DOI:** 10.3390/ijerph23010075

**Published:** 2026-01-05

**Authors:** Micheal Teron Pillay, Mai Thi Quỳnh Le, Yuki Takamatsu, Tran Vu Phong, Nyakallo Kgalane, Noboru Minakawa

**Affiliations:** 1 Institute of Tropical Medicine (NEKKEN), Nagasaki University, Nagasaki 852-8520, Japan; 2 Department of Vector Ecology and Environment, Nagasaki University, Nagasaki 852-8520, Japan; 3 National Institute of Health and Epidemiology, Hanoi 100000, Vietnam; 4 Limpopo Department of Health, Malaria Control, Tzaneen 0850, South Africa

**Keywords:** deep learning, climate–disease interactions, vector-borne disease forecasting

## Abstract

**Highlights:**

**Public health relevance—How does this work relate to a public health issue?**

- Malaria and dengue remain major vector-borne disease burdens whose transmission is strongly shaped by climate variations and forcing.
- These diseases are difficult to detect in real time because clinical case data often lag behind environmental changes.

**Public health significance—Why is this work of significance to public health?**

- The model identifies which climate features become most informative at different prediction horizons, giving public health programs clearer signals about when environmental changes become actionable.
- By extracting climate “risk-profiles” linked to elevated disease incidence, the study helps translate complex environmental information into practical indicators for malaria and dengue surveillance teams.

**Public health implications—What are the key implications or messages for practitioners, policy makers and/or researchers in public health?**

- The study provides concrete temperature and rainfall ranges that typically precede higher malaria and dengue activity, offering clear environmental cues to monitor before cases rise.
- The deep-learning framework shows that routinely available climate variables can support reliable early prediction, improving situational awareness and guiding when to intensify vector-control or preparedness measures.

**Abstract:**

Vector-borne diseases are strongly influenced by climate, yet the magnitude and temporal variability of climate–disease relationships remain poorly quantified. Outbreaks occur abruptly, and responses are typically delayed, underscoring the need for predictive tools that can support proactive interventions. This study applies Temporal Fusion Transformers (TFTs) to long-term, high-resolution climate datasets and to weekly malaria and dengue case records from South Africa and Vietnam to assess predictive performance and identify key environmental drivers. The models incorporated diverse climatic predictors and large-scale climate indices and were trained using multi-horizon forecasting with novel loss functions and physics-based constraints. The best malaria model achieved an R^2^ of 0.95 and an MAE of 4.98, while leading dengue models reached R^2^ values up to 0.90. Variable-importance analyses derived from model-learned weights showed that extreme temperature and rainfall metrics were consistently the strongest predictors, with ENSO (El Niño Southern Oscillation) and IOD (Indian Ocean Dipole) improving longer-range malaria forecasts. Furthermore, climate–disease risk dynamics were explored, revealing specific temperature and rainfall thresholds associated with elevated transmission and highlighting non-stationary relationships across decades. These findings demonstrate accurate, interpretable forecasting offered by TFTs and represent a valuable tool for early warning and understanding of complex climate–disease interactions.

## 1. Introduction

Vector-borne diseases remain a major public health issue affecting millions globally. Malaria and Dengue are especially widespread and problematic diseases that pose significant public health challenges, particularly in tropical and subtropical regions with a heavy burden in Africa [1]. Malaria is responsible for over 600,000 deaths annually, and mortality rates have increased by at least 20% in 19 countries since 2021 [1]. At the same time, dengue has become one of the leading viral vector-borne diseases in terms of global mortality and infectivity, with over 50% of the global population across over 128 countries at risk [2,3]. In the effort to mitigate and decrease the burden of these diseases, early warning has been called for to enable proactive responses to these diseases [1,4].

The ecology between malaria and dengue is similar, as both vectors share analogous relationships with the environment [4]. Dengue is transmitted by *Aedes aegypti* and *Aedes albopictus*. Similarly to malaria, dengue is sensitive to climate as the virus development is linked to the host mosquitoes, whose life cycles are directly influenced by temperature, rainfall, and certainly influenced by other climate factors, including but not limited to humidity [2,5,6,7]. Plasmodium parasites, which cause malaria, are transmitted through the bites of infected female *Anopheles* mosquitoes [8]. Both the parasite and vector are also influenced by multiple climatic variables such as temperature, humidity, and precipitation, which contribute to the incidence and spread of malaria [9,10]. These environmental factors influence both the lifecycle of the parasite and the breeding habitats of the mosquito, thereby affecting the transmission dynamics, such as intensity and seasonality of the disease [11,12]. Since a multitude of climate and environmental data are readily available at high spatial and temporal resolution, it is advantageous to use these data as the core predictors of malaria and dengue incidence in any specified geographic region [13]. While the relationships between climate and these diseases are accepted [7,9,12,14] and their use widespread [13], the specific mechanisms and magnitudes (intervals of risk) of the climate system and different climate variables has not been explored, leaving a gap in the understanding of climate-disease forcing. The variability of climate impacts regarding how small changes in climate variables might significantly alter disease risk is not consistently clear [9]. This includes challenges in understanding how climate-driven changes in one region might differ dramatically from changes in another due to local ecological and environmental conditions [15,16].

For both malaria and dengue diseases, the WHO promotes early warning and prediction as an important intervention for disease control [1,4,17]. Previous studies on dengue and malaria prediction and links between climate have yielded consistent results (Table 1). Temperature and rainfall are usually the key focus of malaria–climate relationships, with multiple studies from species reaction tests in laboratory studies, global climate change modeling studies, and population-based studies all indicating potential climate-related expansion and shifts of vector-borne diseases [10,14,15]. Research has been dedicated to understanding the link between climate variables and the incidence of malaria in order to better predict the disease outcomes. Studies have employed various statistical and machine learning techniques, ranging from logistic regression to random forests, to predict malaria cases based on environmental factors [4,16]. However, these traditional methods often struggle with capturing the temporal dependencies and multivariate complexities inherent in climate and epidemiological data [18]. Recent advancements in the field of disease prediction have seen a notable shift from traditional epidemiological [19] and statistical [4] models toward more sophisticated machine learning techniques [20,21,22], including deep learning [20,22,23,24]. With the advent of deep learning, there is a growing trend to employ more complex neural network architectures, particularly transformers [18,25], which offer significant improvements in handling sequential data and capturing temporal dependencies [26]. This shift is particularly relevant in the context of integrating high-resolution climate data, which have become increasingly accessible and detailed, providing a rich source of predictors for disease outbreaks, especially where the target disease has strong correlations with climate. The use of such advanced predictive models marks a pivotal development in disease forecasting, enabling more accurate and timely predictions that are crucial for effective disease management and prevention strategies.

While there have been applications of deep learning models (Table 1), including transformers, limitations in understanding and verifying the model performance still exists [16,18,25,27]. The application of advanced transformer models, such as Temporal Fusion Transformers (TFTs), has garnered attention in time series prediction due to their ability to handle complex time series data with multivariate dependencies. While the use of TFTs for these diseases has not yet been applied, they have demonstrated significant potential for multi-horizon forecasting and for capturing spatiotemporal dynamics. For instance, TFTs have been explored for anomaly detection in temporal data and were found to effectively identify deviations in complex datasets such as flight data [27], which is conceptually similar to epidemiological anomaly prediction. While not transformer-related, a deep learning model application study by Li et al. [28] indicated early success in fusion-based deep learning approaches for dengue risk forecasting. The use of an adapted loss function and optimization algorithm also helped to improve a deep learning-based prediction model for Dengue [29], however the data used was only up to 2012 and only used rainfall as a climate parameter. In a previous study [18], we also applied the combination of a base transformer and novel loss function, which yielded promising results when trained to predict daily malaria cases; however, only temperature and rainfall were used as predictors. Despite advances in deep learning, current applications have not systematically compared how multi-climate-variable transformer models can improve interpretability and predictive performance for malaria and dengue simultaneously. Hence, the impacts of these variables remain relatively unexplored and unexplained. The application of TFTs in this context is novel and advantageous, potentially offering not only improved prediction accuracy but also the capability to interpret the model and understand which climate variables are most important in influencing malaria and dengue incidence.

**Table 1:** Summary of machine learning and deep learning models applied to malaria and dengue prediction.

Model	Performance	Data	Variables	Author/Source
Machine Learning Model				
ML Classification Algorithms	25–93%	2005–2011	Temperature, RH	Kalipe et al. [22]
WEKA ML Tool and MLP	71%	Monthly Malaria Data	Temperature, RH, Rainfall	Mohapatra et al. [21]
ML Classification Algorithms	80%	1998–2020	Sea surface temperature variability (ENSO, IOD)	Martineau et al. [16]
Fuzzy Classification	PPV = 0.78	Daily Dengue (1993–2011)	Temperature, Rainfall, NDVI, EVI, SST, Socio-economic, Political stability	Buczak et al. [30]
Neural Networks				
Neural Networks (MLP)	72.8%	Monthly Malaria Data (1994–1999)	Temperature, Rainfall, Relative Humidity, NDVI	Kiang et al. [31]
Artificial Neural Networks	82%	Monthly Malaria Data (1995–2014)	Temperature, Rainfall, Relative Humidity, NDVI	Santosh & Ramesh [23]
Radial Basis Function Neural Network	97%	Weekly (1991–2012)	Rainfall	Nanda et al. [29]
Deep Neural Networks (Deep Learning)				
Classifiers/Deep Learning	70.3%	Annual Malaria Incidence (2000–2017)	Rainfall, Temperature	Masinde [32]
LSTM, CNN, Transformer	1.60 (RMSE)	Monthly Dengue (1997–2016)	Temperature, Humidity, Rainfall, Evaporation, Sunshine hours	Nguyen et al. [25]
Deep Learning Transformers	89%; 8.1 (MAE)	1998–2021	Temperature, Rainfall, Cosine time-values	Pillay et al. [18]

Table summarizes various models, performance metrics, datasets, and key environmental variables used in malaria and dengue prediction studies.

In the malaria and dengue system, multiple factors, including the vector, the parasite, and both natural and man-made environmental elements, can influence malaria outcomes [11,33]. These factors consist of multiple elements such as species behavior, parasite resistance, and infection dynamics, human behavior, environmental change, and variability [11,14,33,34,35]. Within this system, the elements have a high degree of variability, which is hard to capture in data or predict [4,10]. Interventions against malaria can themselves interfere with accurate prediction, as the relationship between climate and malaria outcomes may no longer be linear [16,35,36]. The use of a Temporal Fusion Transformer for malaria prediction can offer multiple advantages, as the model is inherently grounded in long- and short-term patterns and associative frameworks within a temporally sensitive environment. While it may be hard to predict every element or sub-element within the malaria system, the nature of deep learning models such as the TFT, which integrate transformers and attention mechanisms, enables robust determinations of relationships between the dependent variable and the predictors even when relationships are very nuanced and subtle [15,37]. Traditional models often struggle with multivariate inputs, long-term dependencies, and the integration of static (time-invariant) and dynamic (time-variant) features [4,16]. It may also be important to understand the magnitude and variable importance of the relationships and interactions between the climate system and disease incidence. Furthermore, in the scope of a changing climate and warming environment, such understanding can be valuable to estimate future changes in vector-borne diseases [9,10] and provide advancement in building an understanding between the complex interactions of climate and health. Using multi-disease datasets with high temporal resolution is rare, and this study will aim to apply such datasets in its analysis, adding to the research and showcasing the possible prediction skill and explainability of deep learning transformers when paired with such data.

The primary objective of this study is to use TFTs to predict malaria and dengue cases in two countries using multiple climate variables and high-temporal-resolution hospital case data. The TFT model has been selected due to its robustness in handling multivariate time-series data and its capability for interpretability. By achieving accurate predictive modeling, we aim to provide a tool that can assist public health officials in planning interventions and allocating resources more effectively. Furthermore, we seek to leverage the interpretability of the TFT to understand the complex relationships between climate variables and disease incidence (malaria and dengue). This will be used to guide the definition of a robust climatology for malaria and dengue and provide common climate–magnitude thresholds at which the risk of case incidence is highest. Additionally, the underlying dynamics and complex interactions between the climate system and disease outcomes will be explored.

Therefore, this study addresses a critical gap in climate–disease modeling by applying the TFT framework to malaria and dengue prediction. By leveraging long-term climate datasets with high temporal resolution, we aim to demonstrate the advantages of transformer-based architectures in capturing complex climate–disease interactions and improving early warning capabilities. The results provide new insights into the relative importance of climatic variables and highlight the potential of interpretable AI approaches for vector-borne disease forecasting under changing climate conditions.

## 2. Materials and Methods

**Figure 1:**
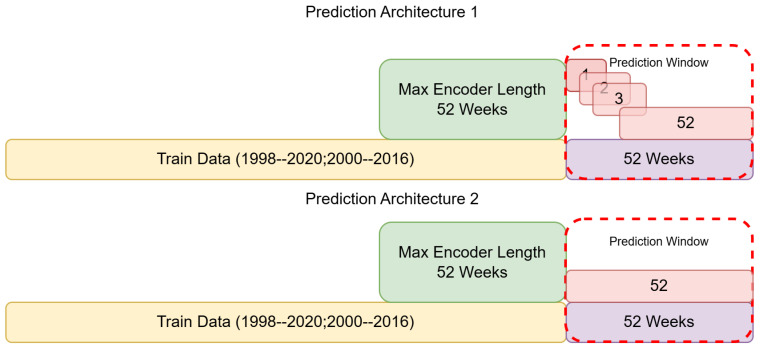
Malaria and dengue case data used, and the splits applied for training and validation. Two different prediction window setups are indicated; prediction architecture 1 indicates a weekly prediction model, where each week from 1 to 52 is predicted separately, and then architecture 2, which indicates a model predicting all 52 weeks at once.

In this study, we explore the application of TFTs to forecast dengue and malaria incidence using time-series data, integrated with multiple climate data. The case incidence datasets consist of daily malaria cases from the Limpopo Health Department. These data are detailed in our previous studies, which assessed plain transformers, statistical, and classical machine learning models, see [16,18,38]. The malaria case data was combined into a weekly timeseries dataset from 1998 to 2021, and monthly dengue cases from the Pasteur Institute covering the period from 2000 to 2018, which was subsequently transformed into weekly data for alignment with the malaria dataset. From each dataset, the last two years of weekly aggregated data (Figure 1) for malaria and dengue (2021, 2022 and 2017, 2018, respectively) were excluded from training and used to test and validate the trained models in order to verify the prediction accuracy and real-world applicability of the model [16,18].

A comprehensive array of climate variables was applied for training the models, including maximum and minimum temperatures (MaxT, MinT), extreme and average rainfall measures (Max_Rain, Min_Rain, rain), potential evapotranspiration rate (PEVR), precipitable water vapor (PWV), relative humidity (RH), soil moisture content (SMC), and wind-related variables (Uwind, Vwind, Wspd). These variables were extracted from the ERA-5 reanalysis dataset https://cds.climate.copernicus.eu/cdsapp#!/dataset/reanalysis-era5-complete [39] (Accessed 30 May 2024) for the Limpopo region in South Africa, averaged over 27–32° E; 25–22° S and Vietnam, averaged over 102–109° E; 8.2–23.4° N. Selection of these variables was iteratively refined based on their collective correlation with and importance to dengue and malaria incidences [40]. From the various combinations of variables tested, the highest correlation set was chosen and applied to the model for training. To capture temporal patterns more effectively, positional encoding through sine and cosine functions of months and weeks was applied to allow the model to distinguish the temporal patterns in the data. This enhances the model’s ability to interpret seasonal variations in disease incidence in relation to climatic changes [18,24].

**Figure 2:**
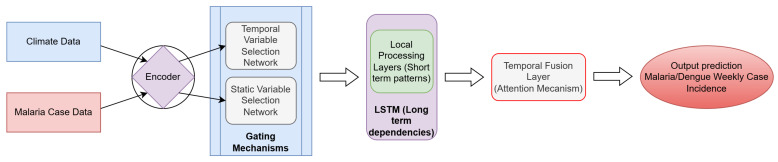
Model architecture with data inputs, throughput processes, and prediction outputs. Refer to Figure A6 for a process-based pipeline indicating the model inputs and outputs.

The TFT model was trained on the preprocessed dataset (Figure 1), partitioned into training, validation, and test sets to ensure the data used to train the model was different from that used to test it, allowing for robust and generalisable predictions and performance results. A rolling window validation strategy was adopted, facilitating the evaluation of the model’s performance over different time windows from 1 to 52 weeks ahead. The training data consists of weekly observations over 22 years, providing a robust dataset for learning patterns and trends. The model (Figure 2) processes these sequences to generate predictions for the subsequent 52 weeks. Two different prediction setups were used: (1) training and running 52 different models for each week to determine which prediction windows were most efficient, and (2) training one model to predict from 1 to 52 weeks. The latter is computationally cheaper and more generalizable but may lack accuracy for the larger prediction windows, as it has to only use the previous year’s data instead of also incorporating the cases predicted from previous weeks. The prediction window was divided into overlapping segments to allow the model to predict one week at a time, iteratively extending to a full year ahead. The previously predicted weeks were each used to update and predict the next week iteratively, so the model could rely on its own predictions to inform the following week to replicate a real-world application when data is not available for the prediction period. This approach ensures that the model can leverage recent patterns and trends effectively, enhancing the accuracy of the predictions.

The predictive performance of the TFT forecasting models was evaluated using four standard regression metrics (see Appendix B for further explanation): Mean Absolute Error (MAE), Mean Squared Error (MSE), Root Mean Squared Error (RMSE), and the Coefficient of Determination ($R^{2}$).

Let $y_{t}$ denote the observed dengue case count at time *t*, ${\hat{y}}_{t}$ the corresponding model prediction, $\bar{y}$ the mean of observed values, and *N* the total number of observations.

Mean Absolute Error (MAE)
  (1) $$\mathrm{MAE}=\frac{1}{N}\sum_{t=1}^{N}\left|y_{t}-{\hat{y}}_{t}\right|$$
Mean Squared Error (MSE)
  (2) $$\mathrm{MSE}=\frac{1}{N}\sum_{t=1}^{N}{\left(y_{t}-{\hat{y}}_{t}\right)}^{2}$$
Root Mean Squared Error (RMSE)
  (3) $$\mathrm{RMSE}=\sqrt{\frac{1}{N}\sum_{t=1}^{N}{\left(y_{t}-{\hat{y}}_{t}\right)}^{2}}$$
Coefficient of Determination (R^2^)
  (4) $$R^{2}=1-\frac{\sum_{t=1}^{N}{\left(y_{t}-{\hat{y}}_{t}\right)}^{2}}{\sum_{t=1}^{N}{\left(y_{t}-\bar{y}\right)}^{2}}$$

Models were trained and run to predict each week from 1 to 52 (a year) to determine which weeks could be predicted and to what accuracy, given the data used. In addition, some data, specifically Niño, DMI, and soil moisture, were removed to assess the model accuracy and stability, as these modes have wide spatial effects and tend to operate at larger timescales and therefore tend to affect the prediction accuracy at smaller timescales [16]. Removing these features also enabled an assessment of their relative importance using the Leave One Covariate Out (LOCO) approach described by Ewald et al. [41]. This was only necessary for malaria predictions, as dengue in Vietnam indicated no such sensitivity, and model retraining did not benefit from removing any variables used. Following this, the top 10 model predictions were shortlisted (if the same model met these criteria more than once, its results were added) based on an R^2^ over 75%. This was done to ensure a representation of the best window to apply these models, as it is difficult to achieve a setup that could predict well for every week of the year based on the climate data alone. Therefore, we can gain a picture of how far the climate data we do have enables us to predict and choose the best model to do so [41,42]. This reflects reality more closely, where real-time setups can be heavily limited to only certain data, in this case, climate data. See Table 1.

In order to determine the specific relationships between climate and disease incidence outcomes, model weights from 100 individual model runs were averaged using a kernel density estimate. This allowed the relationship between variable importance and prediction time steps to be quantified, thereby identifying the optimal prediction window for each variable. Different climate data and variables enable varied predictive power, with some, such as El Niño Southern Oscillation (ENSO), being more reliable for long-term monthly and seasonal predictions [16,42,43], and others, such as temperature and rainfall, promoting predictions at a daily [18] or weekly [4] scale. Following this, the variables with the highest importance, shortlisted based on model importance weights, were used to construct the magnitudes that these variables posed the highest predicted relative risk across lags (2 to 4 weeks and up to 12 weeks based on previous studies [2,4,16,43]) for malaria and dengue. Furthermore, to determine the association between the climate variables and malaria and dengue incidence, relative risk (RR) was calculated for these variables separately [4] and then a compound decadal RR based on multiple variable magnitudes that mapped to the highest RR. This decadal scale was chosen to explore the long-term ‘climatology’ of climate influence on malaria and dengue and how it has changed over time. To account for potential non-stationarity in the relationship between climate variables and disease incidence over time, a moving 5-year window approach was applied. Within each 5-year period, the relative risk (RR) of disease incidence was calculated by dividing the weekly case count by the mean weekly incidence during that specific window. For each window, the combination of temperature and rainfall conditions associated with the highest RR was identified. This allowed an assessment of how climate conditions associated with elevated disease risk shifted over time, independent of long-term trends or short-term fluctuations [38,44]. The RR values and their associated climate magnitudes were then extracted and analyzed across successive 5-year intervals to detect temporal patterns in disease–climate relationships.

## 3. Results

### 3.1. Model Timeseries Predictions

**Figure 3:**
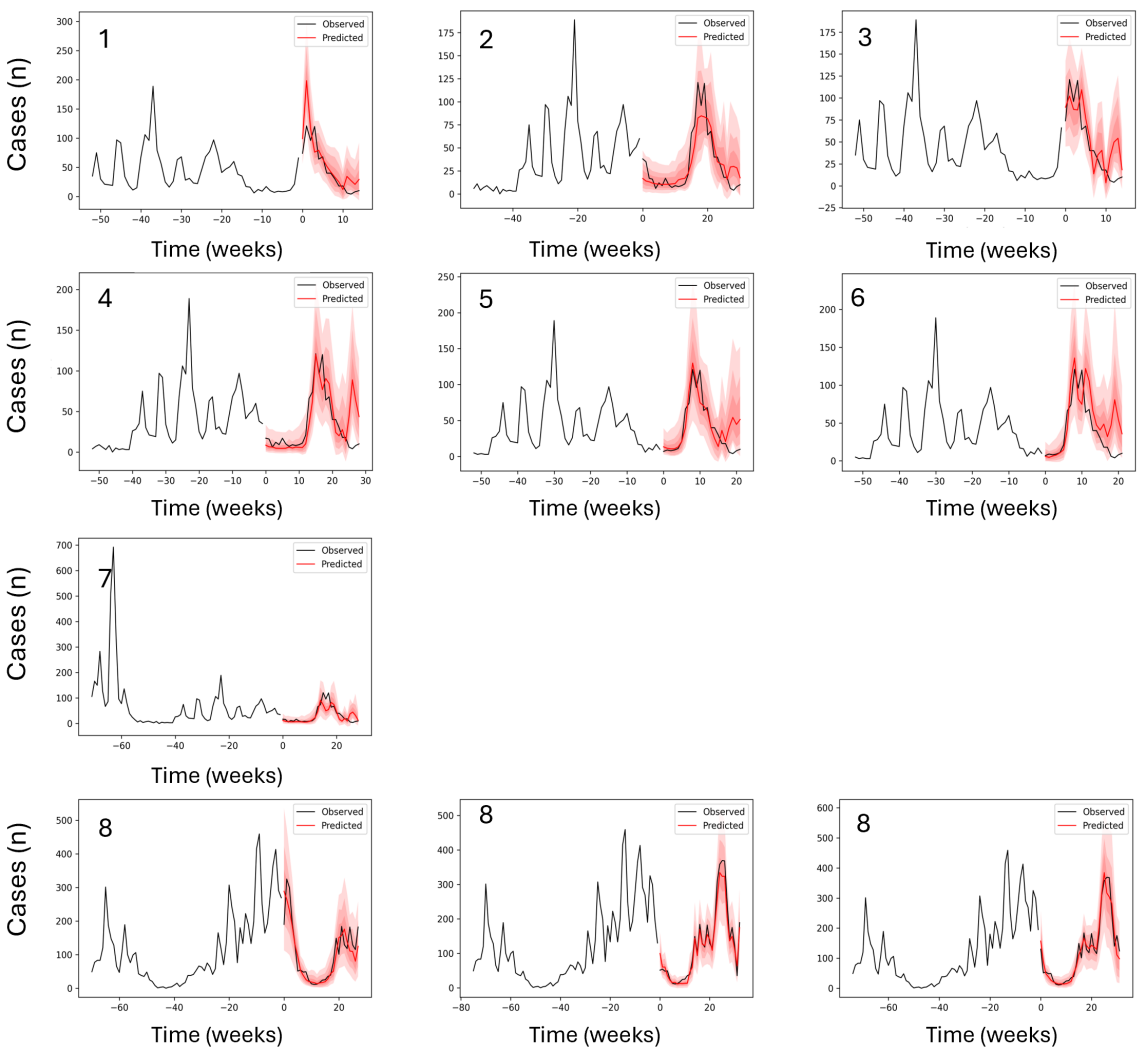
Out-of-sample prediction performance for the top-ranked malaria forecasting models. Each panel represents a trained model, with the panel number corresponding to the model identifier in Table 2. The black line shows observed weekly malaria cases, and the red line shows out-of-sample predictions, with observed cases overlaid during the prediction period. The x-axis indicates time (weeks) and the y-axis shows case counts (Cases [n]). Y-axis ranges vary between panels to reflect differences in case magnitude across time periods. In cases where the same model ranked among the top performers across multiple prediction windows, it appears in more than one panel; therefore, fewer than ten unique models are shown. Full results for all models are provided in Figure A5.

The LOCO method exploration indicated that models without long-term variability features, such as Niño and IOD/DMI, were limited in terms of maximum prediction length compared to the models that did not exclude them (Table 2). The top-performing TFT models for malaria prediction (Figure 3) achieved strong predictive accuracy across varying forecast windows under a weekly prediction regime (see Table 2). The best model, with a maximum encoder length of 100 weeks and a prediction length of 28 weeks, achieved an MAE of 4.98, MSE of 38.66, RMSE of 6.22, and an R^2^ of 0.95. This model retained all input variables and outperformed those in which key predictors were excluded. For instance, when both Niño and smc were removed (Model 2), the MAE increased to 7.32, RMSE to 9.68, and R^2^ dropped to 0.88. Similarly, when Niño, smc, and DMI1 were removed (Model 3), the model produced an MAE of 7.60, RMSE of 11.07, and R^2^ of 0.84. In the most extreme case, removing Niño, smc, and both DMI2 and DMI3 (Model 4) resulted in an MAE of 8.80, RMSE of 12.10, and R^2^ of just 0.83. These results clearly demonstrate the detrimental effect of excluding broader climatic indices, particularly for medium- to long-term forecasts. In contrast, models trained with longer encoder lengths (up to 100 weeks) and without variable exclusions consistently achieved superior performance. The trade-off between model simplicity and predictive power was also evident: while models with reduced input complexity showed acceptable performance, they failed to reach the high R^2^ values observed in models with full variable sets.

#### 3.1.1. Variable Importance for Malaria

**Figure 4:**
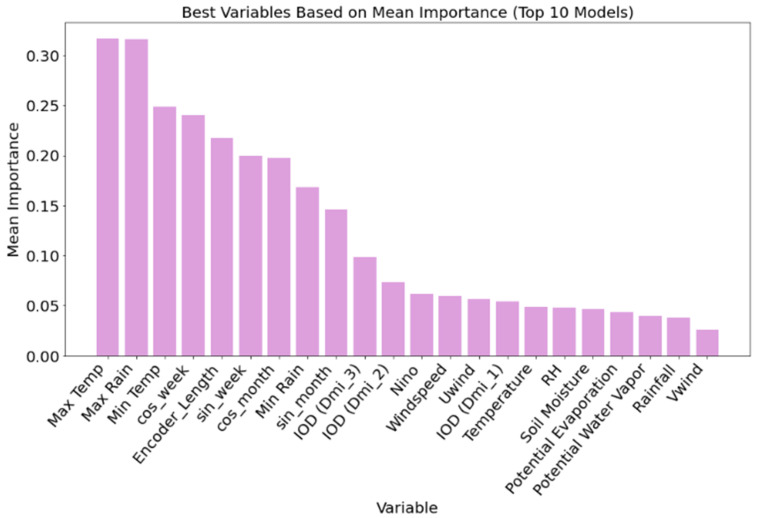
Variable importance based on the combined weights of the top 10 (Figure 3) malaria prediction models for malaria in order of mean importance.

Variable importance analysis, based on the top ten malaria prediction models (Figure 4), revealed that temperature and rainfall-related variables were the most influential predictors. Maximum temperature and maximum rainfall had the highest mean importance scores, each exceeding 0.30, followed closely by minimum temperature and cosine-transformed week-of-year (cos-week), indicating strong seasonality in malaria incidence. Temporal encoding features such as encoder and decoder lengths also ranked highly, underscoring the importance of sequence length and temporal context in accurate forecasting. Soil moisture content and large-scale climate indices, including IOD (Indian Ocean Dipole) components and Niño indices, showed moderate importance, particularly IOD (DMI_3) and IOD (DMI_2), which consistently outperformed Niño in predictive contribution. Other variables, such as relative humidity, potential evapotranspiration, and wind speed, ranked lower, contributing less consistently across models. These results demonstrate that while long-term climate variability plays a role, local and short-term meteorological variables (especially temperature and rainfall) are the most critical drivers of malaria incidence in this modeling framework.

**Figure 5:**
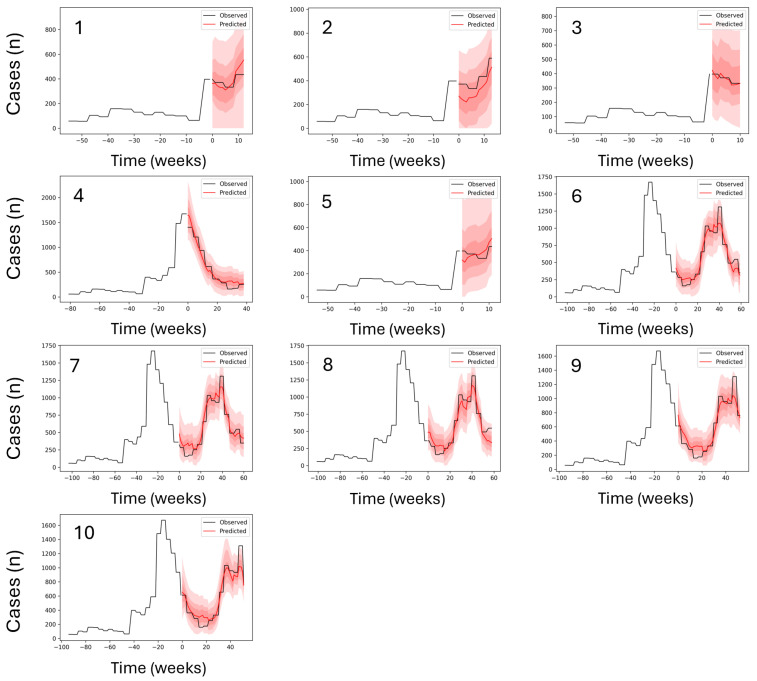
Out-of-sample prediction performance for the top-ranked dengue forecasting models. Each panel represents a trained model, with the panel number corresponding to the model identifier in Table 3. The black line shows observed weekly malaria cases, and the red line shows out-of-sample predictions, with observed cases overlaid during the prediction period. The x-axis indicates time (weeks) and the y-axis shows case counts (Cases [n]). Y-axis ranges vary across panels to reflect differences in case magnitude over time periods.

For dengue predictions, the top-performing TFT models (Table 3, Figure 5) demonstrated robust forecasting accuracy across a wide range of prediction lengths without the need for variable exclusion experiments, as the inclusion or exclusion of broader climate variability indices (e.g., ENSO, IOD) had no discernible effect on model performance in this context (Table 2). The best model, with an encoder length of 102 weeks and a prediction length of 60 weeks, achieved the lowest MAE of 295.32 and an RMSE of 698.80, with a corresponding R^2^ of 0.78. However, the highest overall R^2^ was attained by a model with a shorter encoder length of 55 weeks and a 13-week prediction window, yielding an R^2^ of 0.90, MAE of 377.25, and RMSE of 603.80. Models with encoder lengths between 95 and 103 weeks and long prediction windows of over 50 weeks (Models 6–9) consistently achieved MAEs between 295 and 317, and RMSEs between 698 and 758, with R^2^ values ranging from 0.76 to 0.78, indicating stable performance even at extended horizons. While longer prediction windows slightly reduced R^2^ values compared to shorter-range models, overall accuracy remained high, affirming the model’s ability to generalize well across varying temporal contexts.

#### 3.1.2. Variable Importance for Dengue

**Figure 6:**
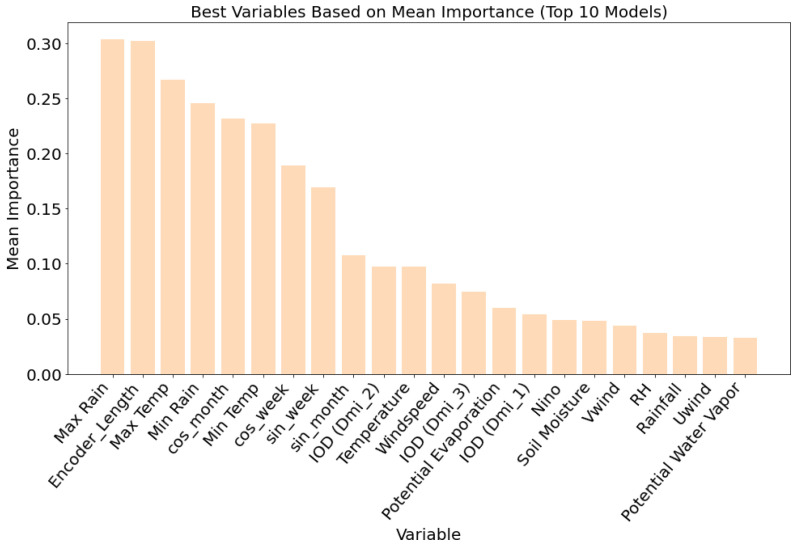
Variable importance based on the combined weights of the top 10 (Figure 5) dengue prediction models for malaria in order of mean importance.

For dengue predictions, variable importance analysis Figure 6 across the top ten models indicated a strong dependence on rainfall and temperature-related variables. Maximum rainfall emerged as the most important predictor, followed closely by encoder length, maximum temperature, and minimum rainfall, each with mean importance values approaching or exceeding 0.25. Temporal features such as cosine-transformed month and week (cos-month and cos-week) and minimum temperature also ranked highly, highlighting the seasonal nature of dengue transmission. While long-term climate variability indicators such as the Dipole Mode Index (IOD) and Niño had comparatively lower importance, variables such as IOD (DMI_2) and IOD (DMI_3) still contributed modestly, suggesting some influence at broader spatial and temporal scales. Features such as soil moisture, relative humidity, wind speed, and potential evapotranspiration had a limited impact on model performance. These findings emphasize the predictive value of localized weather variables and seasonal cycles over global climate indices for dengue forecasting in Vietnam, and further support the suitability of the TFT framework for modeling disease dynamics driven primarily by short-term environmental variability.

#### 3.1.3. Variable Importance and Prediction Length

**Figure 7:**
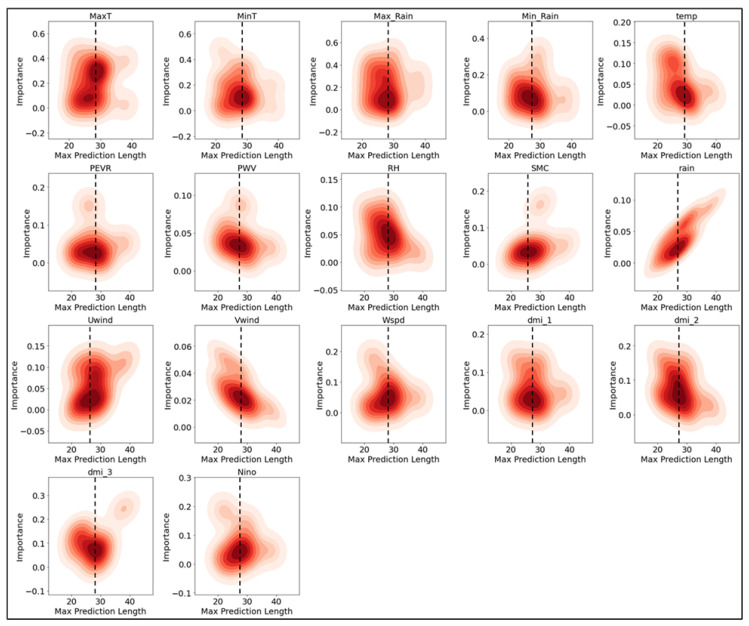
Max prediction length in relation to variable importance across multiple climate variables used as features in the model. Each subplot represents the importance of a specific variable (such as maximum temperature, minimum temperature, rainfall, etc.) against the prediction length. The red hues indicate the saturation of model prediction frequency at a prediction length and importance for each climate variable. Darker hues indicate that a majority of variables were n–magnitude of importance for a certain prediction length. Importance metrics are derived from model weights (see Appendix A).

To determine the importance of various climatic variables across different weekly maximum prediction lengths, the importance data for each variable was aggregated from 100 model runs and presented as density plots (Figure 7). Each subplot represents the importance of a specific variable (e.g., maximum temperature, minimum temperature, rainfall) as a function of prediction length. From the plots, it is evident that certain variables maintain consistent importance across different prediction lengths, while others vary significantly. For instance, MaxT (maximum temperature) and MinT (minimum temperature) show a relatively stable importance throughout the prediction horizons. Variables such as Max Rain and Min Rain also exhibit significant importance across most prediction lengths, highlighting their higher importance in the model’s predictions. Other variables, such as temp, rain, and Niño, show more variability in their importance, suggesting that their influence on the model’s predictions may depend on the specific prediction length. The plots for PEVR (Potential Evaporation), PWV (Precipitable Water Vapor), RH (Relative Humidity), and SMC (Soil Moisture Content) indicate moderate to low importance across the prediction lengths. The density plots do indicate that between 25 and 35 weeks is where most variables would contribute optimally to model predictions, but some variables indicate their usefulness beyond this, displaying a potential application for long-term predictions. Overall, these density plots emphasize the dynamic nature of variable importance in the predictive model, with some climatic variables consistently contributing to the model’s accuracy, while others show fluctuating levels of importance depending on the prediction horizon.

#### 3.1.4. Climate Factors and Disease Dynamics

**Figure 8:**
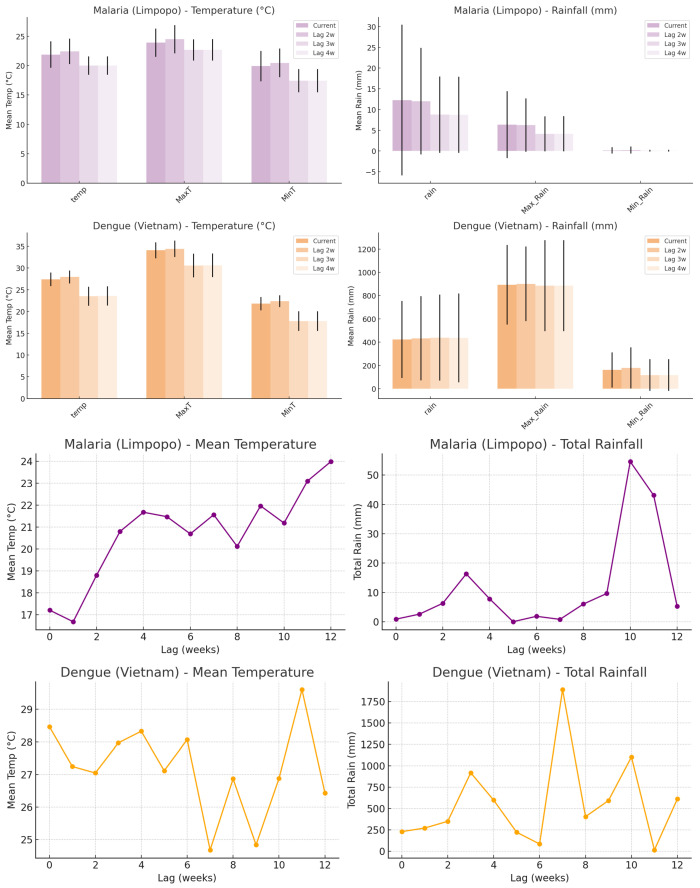
For each lag (0 to 4 weeks), the temperature or rainfall was observed in the week where RR was highest. The mean magnitudes (±1 SD) of three temperature-related variables (mean temperature, maximum temperature, and minimum temperature) and three rainfall-related variables (total rainfall, maximum daily rainfall, and minimum daily rainfall) during these high-risk weeks. The upper purple bars display temperature and rainfall conditions associated with high-risk malaria incidence in Limpopo, while the upper orange bars show those linked with dengue in Vietnam. The lower line graphs (purple) indicate the temperatures and lags in weeks where risk for malaria is highest, while the lower line graph (orange) indicates the temperatures and lags in weeks where risk for dengue incidence is highest.

Based on the model representations of the most important climate factors, temperature and rainfall are consistently highlighted. Hence, temperature, rainfall, and the maxima and minima for each were chosen to explore the climate–disease relationship. These were extracted to understand the model weights (what the model sees as the most likely magnitudes of temperature and rainfall at which risk for case incidence is highest) and inform the climate–disease dynamics. When malaria cases in Limpopo exceeded the 90th percentile, the mean temperature was approximately 22 °C, with maximum and minimum temperatures averaging around 27 °C and 17 °C, respectively. Total weekly rainfall during these high-case weeks averaged approximately 15–20 mm, with moderate maximum daily rainfall and low minimum daily rainfall values. For dengue in Vietnam, high-case weeks were characterized by higher mean temperatures of around 28 °C, with maximum and minimum temperatures averaging approximately 33 °C and 24 °C, respectively. Rainfall levels during high dengue weeks were substantially higher, with weekly totals exceeding 40 mm on average and markedly elevated maximum daily rainfall values. When examining two- and three-week lags (Figure 8), similar climate profiles were observed, with slightly lower temperatures and rainfall magnitudes at the three-week lag, where relative risk (RR) also showed localized peaks. The climate magnitudes were lower. For malaria, the mean temperature was approximately 0.7 °C lower (21.3 °C), and the rainfall was around 13.6 mm. For dengue, the mean temperature was lower too by 0.6 °C (27.4 °C), and the weekly rainfall was approximately 36.9 mm. However, a broader analysis of all weeks revealed that RR was highest at specific climate thresholds (Figure 7): 21.5 °C and 17.5 mm for malaria (RR ≈ 1.27–1.28), and 24.5 °C and 182.5 mm for dengue (RR ≈ 0.98–1.88), as shown by RR curves.

**Figure 9:**
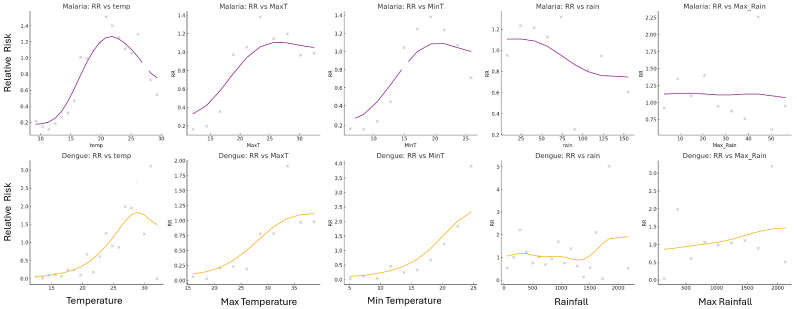
The RR graphs for malaria (purple) and dengue (orange) indicate the temperature, MaxT, MinT, rain, and Max_Rain magnitudes and the corresponding risk for those magnitudes for each disease. Lines are smoothed relative risk, and the points are the raw relative risk data. The peaks of the curves indicate the climate magnitudes associated with the highest RR, thus the highest risk of high disease incidence.

Relative risk patterns associated with temperature and rainfall magnitudes were examined for both malaria and dengue across all weeks. For each disease, smoothed RR curves (Figure 9) were plotted against climate magnitudes to visualize the conditions most associated with elevated case incidence. In Limpopo, the relative risk of malaria was highest at a mean temperature of approximately 22 °C, with RR values peaking sharply and tapering at both lower and higher temperature extremes. Weekly rainfall associated with the highest RR for malaria was around 15 mm, though the curve was broader and less sharply defined compared to temperature. For dengue in Vietnam, the RR was highest at warmer temperatures, peaking near 28–29 °C but remaining high from 30–38 °C maximum temperatures. The RR increased at considerably higher rainfall values, often exceeding 40 mm per week. The smoothed curves illustrated clear peaks, indicating preferred climate ranges where disease transmission risk was maximized. These trends were consistent with earlier findings based on 90th percentile case weeks and confirmed by observing RR trends across multiple climate variable bins.

**Figure 10:**
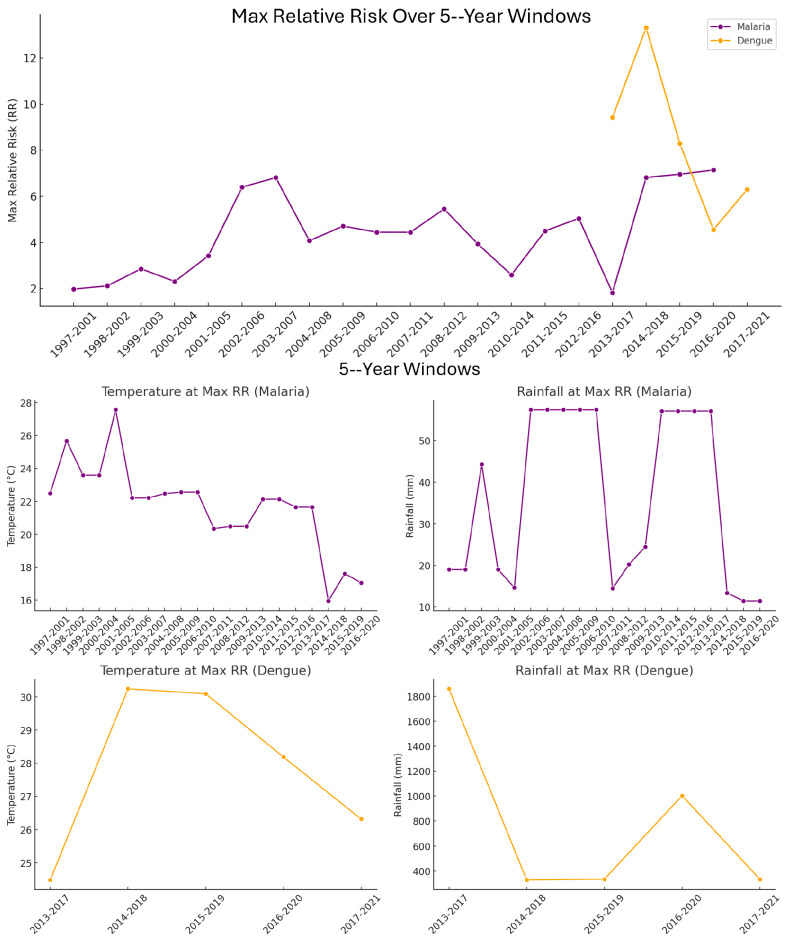
The top panel: Maximum relative risk (RR) of malaria and dengue incidence across moving 5-year windows. Each point represents the highest RR value observed within a given 5-year time window. Purple markers represent malaria cases in Limpopo, South Africa, and orange markers represent dengue cases in Vietnam. The x-axis shows the start and end years of each 5-year window, while the y-axis indicates the corresponding maximum RR value. Temperature and rainfall values associated with the highest relative risk (RR) of malaria and dengue across 5-year moving windows. The middle plots: top row (purple) displays the midpoints of the temperature (°C) and rainfall (mm) bins associated with the highest RR for malaria cases in South Africa. The middle plots bottom row (orange) shows the corresponding metrics for dengue cases in Vietnam. Each data point represents the midpoint of the climate variable bin that corresponded to the highest RR within a given 5-year window, with x-axis labels indicating the start and end year of each window.

The observed temporal variation in climate–disease associations supports the presence of non-stationarity in the relationship between climate variables and disease risk. Specifically, the temperature and rainfall levels associated with the highest relative risk (RR) of malaria and dengue shifted over time (Figure 10), indicating that the influence of climate on disease incidence is not fixed. For malaria, RR peaked at lower temperatures (20–23 °C) and moderate rainfall (20–70 mm), but the exact combination of risk-enhancing conditions varied across decades. In contrast, dengue exhibited consistently higher temperature thresholds (28–30 °C) and substantially higher rainfall (often exceeding 1000 mm) as the climatic conditions most associated with high RR. However, even within dengue data, the precise risk-maximizing temperature–rainfall combination changed slightly between five-year windows. To ensure this non-stationarity was accounted for, RR was also calculated using moving five-year windows, which revealed further shifts in high-risk conditions not captured by broader decadal averages (Table 4).

Decadal analysis of relative risk (Table 4) was performed using joint temperature–rainfall combinations to determine the highest-risk climate profiles associated with malaria and dengue. For each decade, the specific pairing of temperature and rainfall bins that yielded the highest RR was identified. Hence, the values listed in Table 3 do not represent the individual climate factor with the highest isolated risk, but rather the most impactful combination of temperature and rainfall. As a result, optimal climate conditions for elevated disease risk varied between decades. For malaria, peak RR occurred at lower temperatures and moderate rainfall in the 1990s and 2000s, while the 2010s showed a shift to cooler and wetter conditions. In contrast, dengue in the 2010s and 2020s displayed a consistent association with very high rainfall magnitudes and warmer temperatures. These decade-specific shifts suggest that disease risk is driven by the interaction between temperature and rainfall patterns, not by a single climatic variable alone.

## 4. Discussion

Our study demonstrates that the TFT model achieved robust predictive performance for both malaria in Limpopo and dengue in Vietnam, outperforming previously published transformer-based models for vector-borne diseases [16,18,25]. For malaria, the top-performing TFT model achieved an R^2^ of 0.95 and an MAE of 4.98, substantially improving upon the national-level transformer approach we explored in a previous study (see Pillay et al. [18], which reported an R^2^ of 0.84 and an MAE of 8.19 using only rainfall and temperature as predictors. This comparison highlights the combined value of higher-resolution, district-level data and the inclusion of a broader set of climate variables within the TFT framework. Similarly, for dengue, the best-performing TFT model reached an MAE of 295.32 with a 102-week encoder and 60-week prediction length, while a shorter-term model (13-week forecast) achieved the highest R^2^ of 0.90. These results are consistent with Tran et al. [42], who employed the Forecasting Window Transformer for dengue modeling and found that transformers reliably captured multi-week temporal dependencies, particularly when local climatic drivers were incorporated. Another transformer-based model employed for Vietnam’s dengue predictions [25], but at a local level reported MAE ranging from 3.5–10.1, but was outperformed by classical machine learning and was unable to attain good prediction accuracy at a monthly scale compared to the weekly scale employed in this study.

The superior performance of models using extended encoder lengths (up to 102 weeks) across both disease systems reinforces the transformer architecture’s ability to capture long-term dependencies [45] with an advantage over traditional sequence-based models. This supports the design rationale, which optimized attention mechanisms for long-sequence forecasting tasks. Collectively, our findings demonstrate the versatility and efficacy of TFT models in climate-sensitive disease prediction and support their implementation in real-time forecasting systems, especially for public health applications in settings with seasonal or climatically driven transmission dynamics.

The variable importance extracted from the models aids in understanding which variables most strongly promote case incidence and to what extent certain variables are important at different prediction timesteps. Numerous studies [20,21,22,23,46,47] have established temperature and rainfall as the most critical environmental determinants for malaria and dengue transmission dynamics. For malaria, warmer temperatures generally accelerate *Plasmodium* development within mosquito vectors (extrinsic incubation period), while also enhancing mosquito breeding and biting rates [47]. However, excessively high temperatures may reduce mosquito survival. Rainfall influences malaria primarily by creating breeding habitats for *Anopheles* mosquitoes, although excessive rainfall can wash away larvae or reduce mosquito populations through flooding [4]. In our study, both maximum and minimum temperature, as well as maximum rainfall, were among the highest-ranked variables in malaria prediction models, consistent with these findings. Similarly, dengue transmission is highly sensitive to localized weather conditions. Rainfall contributes to the proliferation of *Aedes aegypti* by filling artificial containers and stagnant water sources, providing breeding grounds, while temperature affects mosquito development, virus replication, and transmission potential [48]. Studies have shown that dengue outbreaks often follow periods of increased rainfall combined with favorable temperatures, typically ranging between 25 °C and 32 °C [47,49]. Our variable importance analysis across the top dengue models also highlighted maximum rainfall and temperature as key features.

In both disease systems, the interaction between rainfall and temperature exerts a synergistic effect on vector populations and pathogen development, emphasizing the importance of including these variables in forecasting models. These dynamics are especially critical in regions with seasonal transmission patterns, where even slight shifts in climate variables can influence outbreak timing and magnitude. The consistent prominence of these predictors in our TFT models further underscores their role as important components of climate-informed disease early warning systems. Our variable importance analysis across both diseases confirmed that localized meteorological factors (particularly maximum temperature and rainfall features) are highly influential predictors for malaria and dengue, echoing existing studies [50,51], who demonstrated the dominance of temperature and precipitation in dengue prediction using deep learning. In the case of malaria, the exclusion of broader climate indices such as ENSO and IOD significantly reduced predictive performance, suggesting a supportive but secondary role of these drivers, particularly for long-range forecasts. In contrast, dengue predictions remained robust regardless of global climate variable inclusion, indicating a stronger dependence on short-term, local weather fluctuations, consistent with the ecology of *Aedes* mosquitoes, which are sensitive to rapid changes in temperature and rainfall [49,51,52,53]. These findings emphasize the importance of including long-term climatic drivers in disease forecasting models and validate the use of TFTs for capturing complex interactions between time-dependent environmental factors and malaria incidence.

High risk for dengue incidence was calculated at temperatures from 28–29 °C, with risk remaining even once maximum temperatures exceeded the 35 °C threshold (Figure 9), and indeed it is well known that for dengue vectors in Vietnam, egg-to-pupae development increases as temperatures rise from 12 to 30 °C and only decreases after temperatures pass 40 °C [25]. Furthermore, it has also been observed that the temperature window around 32–33 °C is optimal for endemic transmission and incidence rate [46,52]. Sustained rainfall and temperature between 25–32 °C were also followed by increased cases in Singapore [47,53,54]. Liu-Helmersson et al. [48] specifically found that average temperature up to 29 °C increased dengue epidemic potential, which incidentally decreased at magnitudes higher than this. Our climate risk analysis has been able to capture these high-risk intervals of temperature, indicating the value of their use in the prediction models and confirming that they indeed reflect that the model is capturing the correct high-risk temperatures and assigning the correct weights when predicting. These patterns help characterize the climate conditions most strongly associated with elevated transmission risk. This is valuable because the prediction is not the most important output, but also the associative understanding of the relationship between disease incidence and climate.

Although rainfall and temperature consistently emerge as the dominant predictors of malaria and dengue incidence, the predictive performance of the model depends on the inclusion of a broader set of climate variables. These additional inputs allow the model to account for background climate variability, reduce noise, and learn interactions across timescales. While short-term forecasts are largely driven by local temperature and rainfall, longer-range predictions benefit from large-scale climate modes such as ENSO and the Indian Ocean Dipole, which exert stronger influence at seasonal timescales. Importantly, improvements in predictive accuracy should not be interpreted as increasing the biological likelihood of future outbreaks, but rather as reducing uncertainty in how known environmental constraints translate into disease risk. Consequently, the primary biological value of this approach lies not in deterministic long-term prediction but in identifying climate thresholds, lag effects, and periods of elevated risk that can inform surveillance and intervention timing. From a biological perspective, this enables the model to be used not as a black-box predictor, but as a tool for identifying environmentally driven windows of heightened transmission risk that align with known vector and pathogen dynamics. As with all climate-based forecasting systems, performance is constrained by the reliability of climate inputs, which are most robust at seasonal horizons; thus, the framework is best suited for early warning and scenario-based risk assessment rather than precise long-term outbreak prediction.

TFTs are adept at implicit feature capture, identifying hidden patterns in the data even when those patterns are not explicitly labeled. This feature is particularly useful for understanding the underlying structures within the malaria system. While TFTs excel at inferring patterns, it is worth noting that they are not infallible. For instance, if an impactful factor such as human intervention through spraying insecticides is missing from the training data, the model might learn an incomplete or incorrect representation of the system [37,55]. However, by incorporating physics-informed limits that indicate specific intervals of climate data associated with higher malaria risk, the model is less likely to misattribute effects and instead will consider unattributed patterns as a form of floating predictor [55]. This allows the model to focus its attention on the most relevant parts of the data, leveraging attention mechanisms to optimize predictions in the complex, multifaceted environment of malaria transmission.

This strategy underscores the flexibility and generalizability of the TFT model, demonstrating its potential as a global framework for infectious disease forecasting. Our results indicate that the integration of detailed climate data with advanced machine learning techniques, such as TFTs, offers a promising avenue for enhancing the predictability of disease outbreaks, thereby contributing to more effective public health interventions and disease control strategies. The novelty of the TFT model lies in its ability to provide detailed insights into which inputs are influencing the forecasts at any given time, thereby not just offering predictions but also enhancing our understanding of the underlying dynamics of the system being modeled.

A key innovation of this research lies in the application of a singular model architecture to analyze both malaria and dengue fever cases, despite the differences in disease characteristics and geographic contexts. The use of TFTs in this study is particularly novel, leveraging the model’s capability to handle multivariate time-series data with variable selection mechanisms and temporal attention, allowing for the ability to model complex interactions between variables over time. This approach allowed for an in-depth analysis of the interplay between climate variables and disease incidence, highlighting the importance of specific climatic factors in malaria and dengue disease prediction.

## 5. Conclusions

This study demonstrates that Temporal Fusion Transformers offer a robust and interpretable approach for forecasting climate-sensitive vector-borne diseases using routinely available climate data. Applied to long-term weekly malaria and dengue records from South Africa and Vietnam, the models achieved high predictive accuracy across multiple forecast horizons, with malaria predictions reaching an R^2^ of 0.95 and dengue predictions up to 0.90. The TFT framework consistently identified temperature and rainfall—particularly their extreme values—as the most influential drivers of transmission, while large-scale climate indices such as ENSO and the Indian Ocean Dipole improved longer-range malaria forecasts. By coupling strong predictive performance with transparent variable-importance estimates and climate risk profiling, this approach provides both reliable early warning capability and actionable insight into climate–disease relationships. These findings highlight the value of transformer-based models as practical tools to support anticipatory public-health planning, surveillance, and targeted intervention strategies in regions vulnerable to malaria and dengue.

## Figures and Tables

**Table 2:** Top 10 malaria models (weekly model prediction regime) with each model’s max encoder length and prediction length in weekly units and the model accuracy parameters; MAE, MSE, RMSE, and R^2^. The models that were tested with variables such as Niño, DMI (1, 2, and 3), and smc are indicated if applicable.

Model	Max Encoder Length	Max Prediction Length	MAE	MSE	RMSE	R^2^	Variables Removed
1	52 (Fixed)	15	8.21	92.39	9.61	0.88	Niño
2	52 (Fixed)	31	7.32	93.61	9.68	0.88	Smc, Niño
3	52 (Fixed)	15	7.60	122.46	11.07	0.84	Niño, Smc, Dmi1
4	52 (Fixed)	29	8.80	146.50	12.10	0.83	Niño, Smc, Dmi2,3
5	52 (Fixed)	22	6.98	111.26	10.55	0.86	0
6	52 (Fixed)	25 (Sensitive RR)	9.71	130.19	11.41	0.85	0
7	71 (100)	29	8.81	140.40	11.85	0.83	0
8	100 (Fixed)	28	4.98	38.66	6.22	0.95	0
9	100 (Fixed)	33	7.25	89.17	9.44	0.88	0
10	100 (Fixed)	30	9.66	136.66	11.69	0.84	0

Models evaluated under varying encoder and prediction lengths. Variables removed indicate excluded climatic indices.

**Table 3:** Comparison of the top 10 dengue forecasting models under a weekly prediction regime. The table reports the maximum encoder length, prediction horizon (weeks), and corresponding performance metrics (MAE, MSE, RMSE, and R^2^) for each model.

Model	Max Encoder Length	Max Prediction Length	MAE	MSE	RMSE	R^2^
1	55	13	377.25	364,574.00	603.80	0.90
2	56	14	462.04	463,786.00	681.02	0.88
3	53	11	526.18	490,304.80	700.22	0.85
4	81	39	381.92	655,210.90	809.45	0.78
5	54	12	642.24	785,622.40	886.35	0.78
6	102	60	295.32	488,314.50	698.80	0.78
7	103	61	300.36	497,894.40	705.62	0.77
8	101	59	317.82	530,361.40	728.26	0.76
9	95	53	313.07	574,283.80	757.82	0.76
10	74	32	467.33	843,058.40	918.18	0.76

MAE, MSE, RMSE, and R^2^ metrics are reported for each model configuration.

**Table 4:** Decadal risk based on the maximum average risk at specific climate conditions based on temperature and rainfall combined association calculations.

Decade	Dengue			Malaria		
	Temperature ( °C)	Rainfall (mm)	Max RR	Temperature ( °C)	Rainfall (mm)	Max RR
1990	–	–	–	20.4	48	2.96
2000	–	–	–	28.4	61	4.04
2010	29.6	1821	11.04	17.2	79	4.45
2020	28.8	923	8.45	20.5	22	3.12

**Figure A1:**
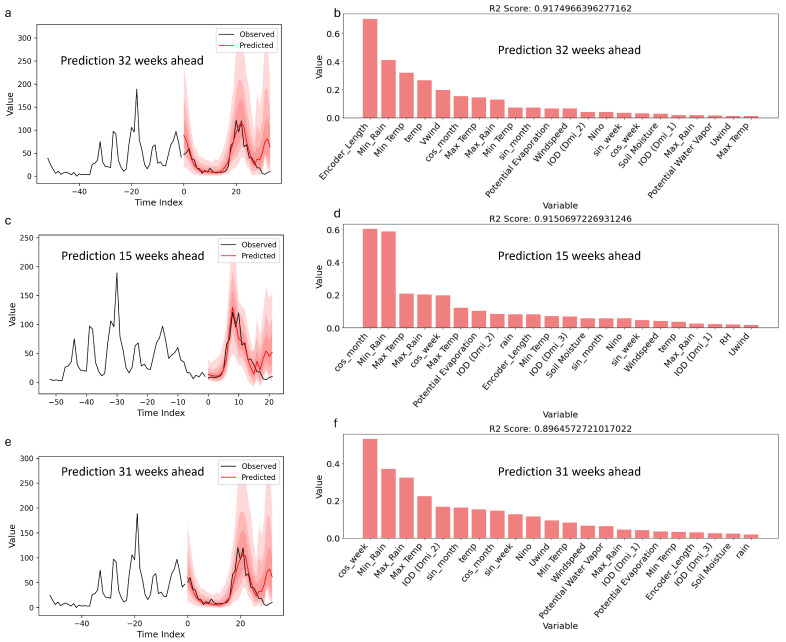
Top three weekly inference-based predictions using the best model for malaria prediction. Predictions in panel (**a**,**c**,**e**): Black lines indicate the observed cases and red the predictions. Variable importance panels (**b**,**d**,**f**) corresponding to (**a**,**c**,**e**) respectively indicate the variable and their magnitude of importance for the model predictions.

**Figure A2:**
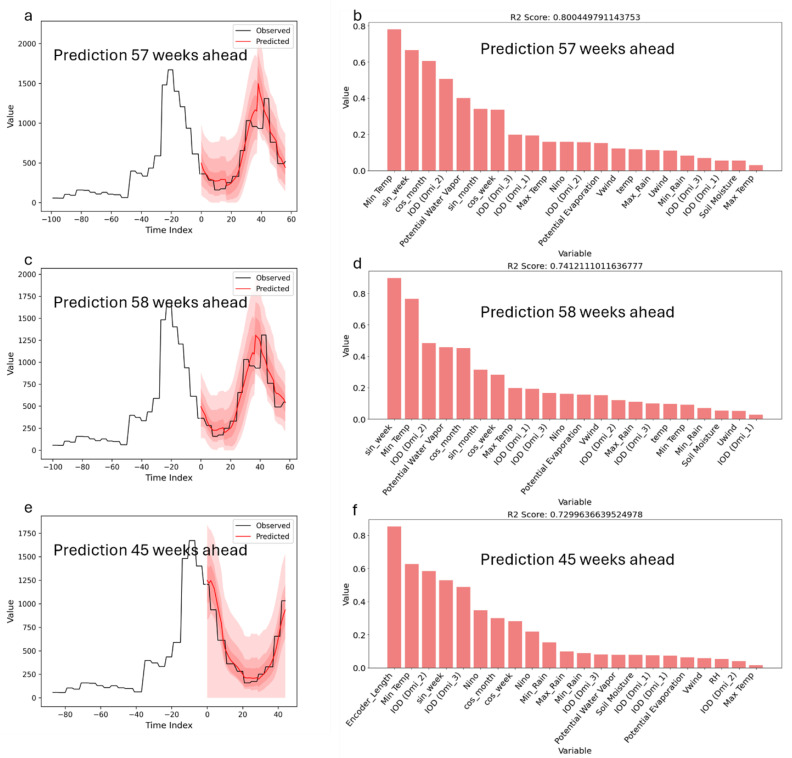
Top three weekly inference-based predictions using the best model for dengue prediction. Predictions in panel (**a**,**c**,**e**): Black lines indicate the observed cases and red the predictions. Variable importance panels (**b**,**d**,**f**) corresponding to (**a**,**c**,**e**) respectively indicate the variable and their magnitude of importance for the model predictions.

**Figure A3:**
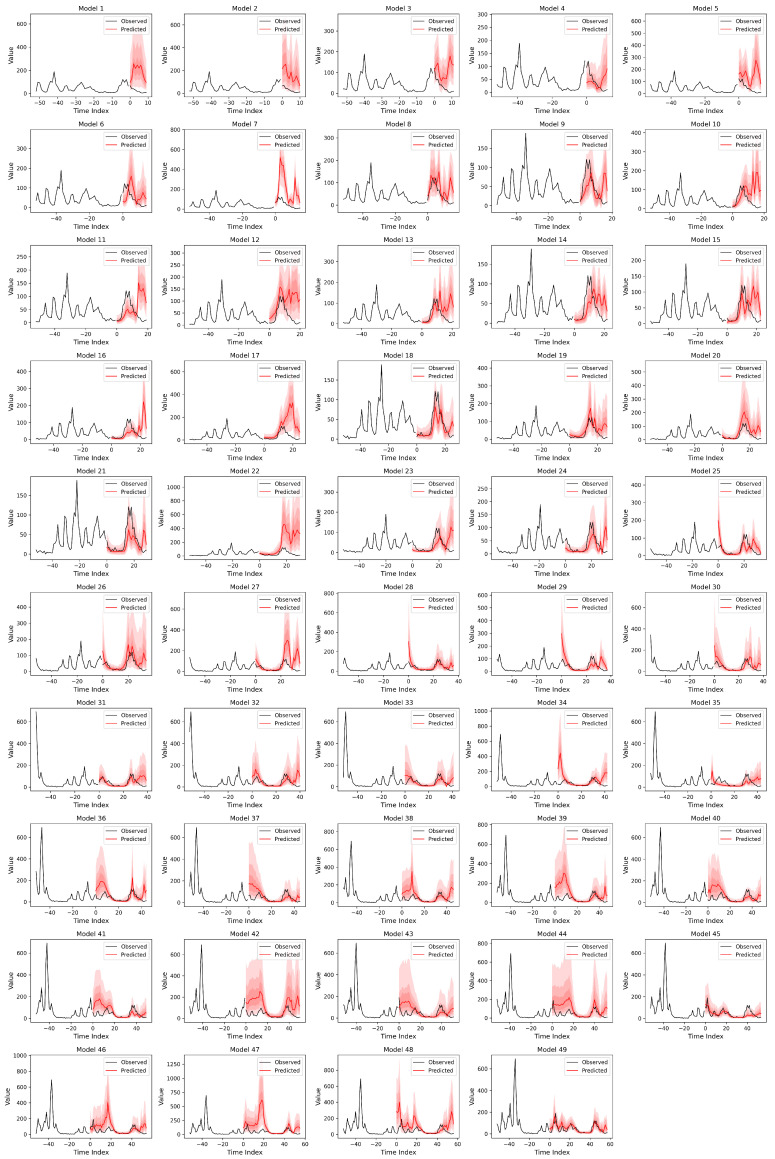
Weekly Malaria Model Predictions week 10 to 60. Using temperature, rainfall, Niño, and DMI, this model achieved over 80% accuracy. Black lines indicate the observed cases and red the predictions.

**Figure A4:**
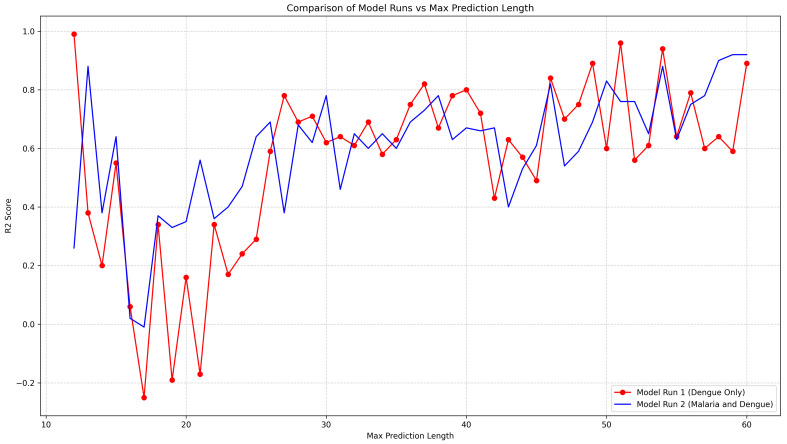
The R^2^ scores for each prediction length are plotted, highlighting the differences in predictive accuracy between the two models, one just using dengue data and the other malaria and dengue data together.

**Figure A5:**
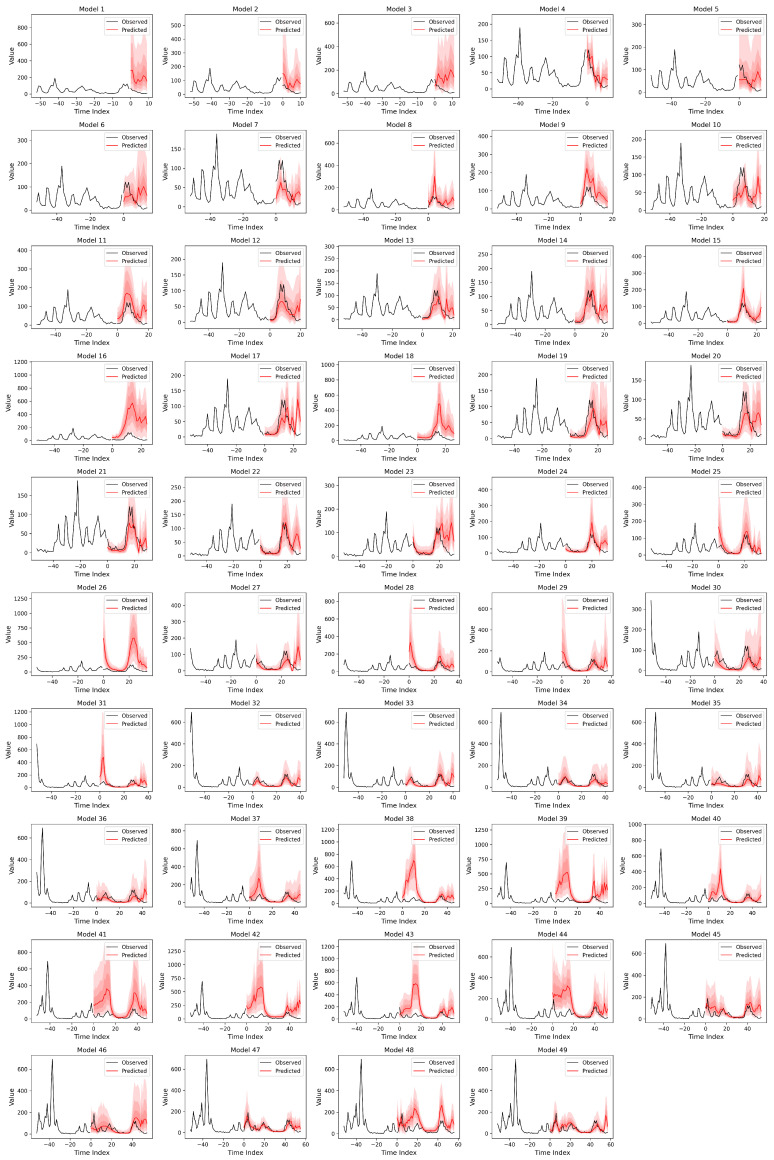
Weekly Malaria Model Predictions week 10 to 60. Using a dynamic RR loss function and removing Niño and Soil Moisture Content, achieved about 88%. Better at longer prediction windows. Black lines indicate the observed cases and red the predictions.

**Figure A6:**
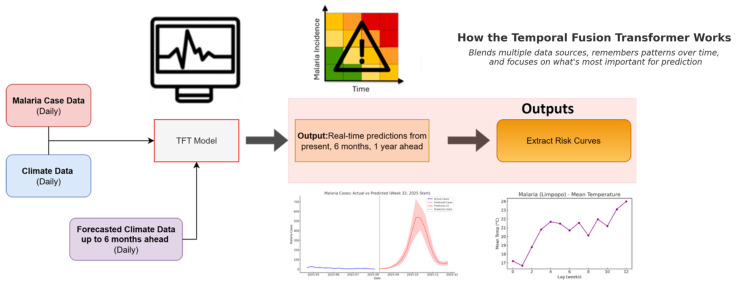
Process-based pipeline including input cases, climate, and predicted climate data and outputs from timeseries predictions seen by red line, to risk curves per time lag depicted in purple. The output graphs depicted her a just a representative sample and do not reflect the actual prediction outputs.

## Data Availability

Case data is dependent on the country of origin’s data sharing policies, and requests will have to be made directly with their health department’s point of contact. Climate data and the model API can be made available on request. The model code can be found on github: https://github.com/MichealTeron/Temporal-Fusion-Transformers-for-Climate-Related-Infectious-Diseases accessed on 7 May 2025.

## Appendix A. Model Implementation and Structure

The TFT model was specifically chosen based on the following mechanisms: 1. Gating mechanisms: These allow the model to manage the flow of information, enabling it to disregard irrelevant data points and focus on those most informative for the prediction task. 2. Variable selection networks: These networks identify and prioritize the most relevant input features at each time step, enhancing model interpretability and efficiency by focusing on key predictors. 3. Temporal attention mechanisms: By weighing the importance of different time steps differently, the model can capture long-term dependencies and relationships within the time series, accommodating patterns such as seasonality or trend shifts. 4. Static covariate encoders: These are used to incorporate static information that affects the time series but does not change over time, such as geographic location or seasonal components. This was specifically used to incorporate and model two distinct diseases from two different countries. 5. Adaptability: The model framework allows flexible modification so that the setup and architecture can be modified to suit the prediction problem and data being used. In this case, attention [26] and physics-based constraints are employed in a novel training method in this paper. For a specific architecture and explanation of the TFT, refer to Lim et al. 2021 [37].

### Appendix A.1. Model Training and Application

The training methodology for our Temporal Fusion Transformer (TFT) model follows a structured approach to optimize its ability to forecast disease incidence rates based on climate variables. Initially, the dataset is segmented into small batches, a technique known as batch training, facilitating efficient optimization by sequentially updating the model’s weights. The discrepancy between the model’s predictions and the actual case data is quantified using a suitable loss function [15,27], such as mean squared error (MSE), but in this case, a custom loss function was developed and applied to deal with the use of two different disease datasets, which had various problems, such as imbalance and non-continuous segments. The optimization of model weights is conducted through the Adam optimization algorithm, which is selected for its effectiveness in minimizing the loss function while also incorporating mechanisms to prevent overfitting, including the adjustment of learning rates and the application of regularization techniques [27]. A dropout of 0.26 was also used to further prevent overfitting of the models.

Following the initial training phase, systematic hyperparameter tuning is performed to identify the optimal model configuration. This process involves adjusting parameters such as the learning rate, batch size, and the number of layers to minimize prediction error on the validation set. Hyperparameters were adjusted until the best model setup was attained: learning rate = 0.0328, hidden size = 30, attention head size = 2, dropout = 0.2658, hidden continuous size = 14, output size = 7, loss = Custom Loss(physics_coeff = 0.1). A portion of the dataset is reserved as a validation set, enabling the fine-tuning of model parameters and the implementation of early stopping based on validation set performance, thus averting overfitting. Model performance is then rigorously evaluated through out-of-sample testing, ensuring the model’s generalization to unseen data. This evaluation compares predicted disease incidences with observed occurrences, using the metrics root mean squared error (RMSE) and mean absolute error (MAE). Multiple models, each with varying configurations and forecast windows, are tested to ascertain the best-performing model.

### Appendix A.2. Novel Additions to the Model Architecture

Physics-based constraints were calculated, and a module was added to the model to process and use these constraints to guide the model’s training process. This higher dimensionality and the inference of underlying structures in the data allow this class of deep learning models to pick up information on many patterns in the data, even if those patterns are not provided explicitly through the data. While such abilities can have disadvantages, such as incorrectly correlating a predictor to a pattern in the data, this paper attempts to circumvent such problems using novel training mechanisms, such as constraints and tailored loss functions.

### Appendix A.3. Standalone Model Predictions and Performance (Top 3)

The best Temporal Fusion Transformer (TFT) model provided predictions for disease incidence at lead times of 57, 58, and 45 weeks, with corresponding R^2^ scores of approximately 0.80, 0.74, and 0.73. The observed and predicted values demonstrated a good alignment, with prediction intervals represented by shaded regions, capturing the uncertainty associated with these forecasts. Variable importance metrics indicated that minimum temperature, sine transformations of the week index (sin_week), and dimensions of the Indian Ocean Dipole (IOD, Dim-2) had the highest contributions to the model’s predictions at 57 weeks. For the 58-week prediction, key variables included sin_week, minimum temperature, and IOD-related indices. At a 45-week horizon, encoder length, minimum temperature, and multiple IOD dimensions were among the top influential factors. These results identify the specific variables that the TFT model considered significant for forecasting disease incidence across varying time horizons.

**Table A1:** Regression accuracy metrics comparing the performance of a previously published transformer model (Pillay et al. [18]) with the Temporal Fusion Transformer (TFT) used in this study. Metrics include explained variance, maximum error, mean absolute error (MAE), and coefficient of determination (R^2^), based on mean aggregated predictions. The reported values reflect model performance at the national scale [18] and the district level (see Figure A2 for the respective transformer implementations).

Regression Accuracy Metrics	Pillay et al., 2023 [18]	Temporal Fusion Transformer	TFT District Level
Explained variance	0.87	0.93	0.99
Max error	40.94	25.60	11.84
MAE	8.19	6.84–10.62	>1
$R^{2}$	0.84	0.81–0.92	0.99

## Appendix B. Model Accuracy Metrics Explanation Extended

Model evaluation metrics. Model performance was evaluated using the mean absolute error (MAE), mean squared error (MSE), and root mean squared error (RMSE), which quantify the average difference between predicted and observed case counts. MAE represents the average absolute error in the original units (cases), while MSE and RMSE penalize larger errors more strongly and are therefore more sensitive to occasional large deviations. Differences in the magnitude of these metrics between malaria and dengue models are expected and primarily reflect differences in the underlying case distributions, including overall incidence levels, variability, and outbreak intensity. Models trained on datasets with higher case counts or greater temporal variability will naturally yield larger absolute error values, even when relative predictive performance is comparable. Consequently, these metrics are most informative when used to compare models within the same disease and dataset rather than across different diseases.
